# Stimulation of Neural Stem Cell Proliferation by Inhibition of Phosphodiesterase 5

**DOI:** 10.1155/2014/878397

**Published:** 2014-01-12

**Authors:** Ana I. Santos, Bruno P. Carreira, Rui J. Nobre, Caetana M. Carvalho, Inês M. Araújo

**Affiliations:** ^1^Centre for Neuroscience and Cell Biology, University of Coimbra, 3004-517 Coimbra, Portugal; ^2^Department of Biomedical Sciences and Medicine, University of Algarve, 8005-139 Faro, Portugal; ^3^Institute for Biotechnology and Bioengineering (IBB), Centre for Molecular and Structural Biomedicine, University of Algarve, 8005-139 Faro, Portugal

## Abstract

The involvement of nitric oxide (NO) and cyclic GMP (cGMP) in neurogenesis has been progressively unmasked over the last decade. Phosphodiesterase 5 (PDE5) specifically degrades cGMP and is highly abundant in the mammalian brain. Inhibition of cGMP hydrolysis by blocking PDE5 is a possible strategy to enhance the first step of neurogenesis, proliferation of neural stem cells (NSC). In this work, we have studied the effect on cell proliferation of 3 inhibitors with different selectivity and potency for PDE5, T0156, sildenafil, and zaprinast, using subventricular zone-(SVZ-) derived NSC cultures. We observed that a short- (6 h) or a long-term (24 h) treatment with PDE5 inhibitors increased SVZ-derived NSC proliferation. Cell proliferation induced by PDE5 inhibitors was dependent on the activation of the mitogen-activated protein kinase (MAPK) and was abolished by inhibitors of MAPK signaling, soluble guanylyl cyclase, and protein kinase G. Moreover, sildenafil neither activated ERK1/2 nor altered p27^Kip1^ levels, suggesting the involvement of pathways different from those activated by T0156 or zaprinast. In agreement with the present results, PDE5 inhibitors may be an interesting therapeutic approach for enhancing the proliferation stage of adult neurogenesis.

## 1. Introduction

Neurogenesis is the biological process of generating new neurons from progenitor cells or neural stem cells (NSC). NSC proliferate in two main regions of the adult mammalian brain: the subventricular zone (SVZ) of the lateral ventricles and the subgranular zone (SGZ) of dentate gyrus of the hippocampus. Following brain injury, such as stroke, NSC in the endogenous niches proliferate and migrate to the affected brain areas where they may differentiate into neurons, but survival is limited [1–3]. There is still a lack of knowledge concerning the use of effective therapeutic strategies in order to overcome the limited ability of brain self-repair following an insult. Understanding the signaling pathways involved in the regulation of neurogenesis is paramount in order to enhance brain repair.

Neurogenesis is affected by several factors, including nitric oxide (NO). NO is a free radical of particular interest due to its cellular function as a second messenger, which includes the regulation of NSC proliferation. Several studies recently reported the effect of NO on the stimulation of adult neurogenesis in the dentate gyrus and in the SVZ [4–8]. Thus, the increase of NO levels following brain injury, such as seizures or ischemia, has been shown to promote proliferation of NSC and the formation of new neurons [4, 5, 8]. Two distinct pathways seem to be involved in the proliferative effect of NO, the extracellular signal-regulated kinase (ERK)/mitogen-activated protein kinase (MAPK) pathway [5] and the soluble guanylyl cyclase (sGC)/protein kinase G (PKG) pathway [9]. The second messenger cyclic GMP (cGMP) is a signaling molecule whose levels are regulated by an equilibrium between its production and removal. cGMP is produced by sGC, which can be activated by NO. Activation of sGC by NO leads to increased cGMP levels that activate downstream targets, such as PKG [10, 11]. PKG is a serine/threonine cGMP-dependent kinase that regulates the activity of several transcription factors that control important processes like synaptic plasticity (reviewed by [12]). In several conditions, such as aging, cGMP levels are decreased and may be involved in age-related neurodegeneration, decreased neurogenesis, and cognitive decline [13]. Similar to the increase of cGMP production by NO, the inhibition of cGMP hydrolysis, by targeting phosphodiesterases (PDE), could be a strategy to increase the levels of cGMP and, consequently, reverse these effects by stimulating neurogenesis.

PDE are ubiquitous enzymes responsible for the degradation of cyclic nucleotides, whose activity is dependent on substrate, kinetic properties, and cellular and subcellular distribution of the 11 known families. PDE5, 6, and 9 are considered cGMP specific PDE, whereas PDE4, 7, and 8 mainly hydrolyze cyclic AMP (cAMP) and PDE1, 2, 3, 10, and 11 hydrolyze both substrates (reviewed in [14]). Phosphodiesterase type 5 is specific for cGMP degradation and is present in the mammalian brain [15, 16]. PDE5 inhibitors have been used for the treatment of several pathologies in which an increase in cGMP levels can be beneficial, such as erectile dysfunction and pulmonary hypertension (reviewed by [17, 18]). The best characterized inhibitor of PDE5 is sildenafil. Sildenafil was first used for the treatment of erectile dysfunction. Due to the presence of PDE5 in the lung, sildenafil is also used in the treatment of pulmonary hypertension (reviewed by [18–20]). Since PDE5 is also present in the central nervous system, several works have assessed the effect of sildenafil on the brain (reviewed by [21]). Some studies reported that sildenafil improves learning and memory [22, 23] and also induces neurogenesis in the SVZ and SGZ [24]. Neuronal function recovery in adult young rats and in aged rats [25], following stroke, has also been described after sildenafil treatment [26, 27]. Zaprinast, the first PDE5 inhibitor to be used, has been shown to be a nonpotent and nonselective inhibitor. Sildenafil is typically considered a PDE5 inhibitor, with an IC_50_ of approximately 4 nM, and it has been shown to inhibit PDE5 at concentrations about 200-fold lower than the concentration of zaprinast required to inhibit PDE5 [28]. However, it also inhibits PDE1 and PDE6 (IC_50_ values of 281 and 29 nM, resp. [28]), indicating that sildenafil is not totally selective for PDE5. More recently, a new compound was developed, T0156, which potently inhibits PDE5 [29]. In fact, T0156 has a much lower IC_50_ for PDE5 (0.23 nM) than sildenafil (IC_50_ 3.6 nM).

Within this scenario, in this work we investigated the effect of three different PDE5 inhibitors: T0156, sildenafil, and zaprinast, on the first step of neurogenesis, by evaluating proliferation of SVZ-derived NSC, and addressed the mechanisms underlying these effects. Here we show that T0156, sildenafil, and zaprinast increase SVZ-derived NSC proliferation. Moreover, blocking MAPK, sGC, or PKG, independently, results in loss of the proliferative effect of PDE5 inhibitors, pointing out to the involvement of sGC/PKG and ERK/MAPK pathways in the stimulation of NSC proliferation by PD5 inhibition.

## 2. Methods

### 2.1. Materials

Dulbecco's Modified Eagle's Medium: F-12 nutrient mixture, (D-MEM/F-12, with GlutaMAX I), B27 supplement, trypsin-ethylenediaminetetraacetic acid (EDTA) solution, antibiotics (10,000  units/mL penicillin, 10 mg/mL streptomycin), Click-iT EdU Alexa Fluor 488 Flow Cytometry Assay kit, Click-iT EdU Alexa Fluor 594 HCS Assay kit, and StemPro Accutase cell dissociation reagent were purchased from Invitrogen (Paisley, UK). Epidermal growth factor (EGF) and basic fibroblast growth factor (bFGF) were from PeproTech Inc. (London, UK). The Matrix used was from Stoelting Co. (Wood Dale, IL, USA). Bicinchoninic acid (BCA) Protein Assay kit was from Pierce (Rockford, IL, USA). 8-bromoguanosine 3′,5′-cyclic monophosphate (8-Br-cGMP), Phenylmethylsulfonyl fluoride, orthovanadate, chymostatin, leuptin, antipain, pepstatin A, trypan blue, Tween-20, mouse anti-*α*-tubulin, 2-Propanol, chloroform, and TRI Reagent were purchased from Sigma Chemical (St Louis, MO, USA). T0156 hydrochloride, sildenafil citrate, zaprinast, and 1*H*-[1,2, 4]Oxadiazolo[4,3-a]quinoxalin-1-one (ODQ) were obtained from Tocris Bioscience (Bristol, UK). KT5823 was obtained from Alomone Labs (Jerusalem, Israel) and 3-(5′-Hydroxymethyl-2′-furyl)-1-benzylindazole (YC-1) from Santa Cruz Biotechnology (Santa Cruz, CA, USA). 1,4-diamino-2,3-dicyano-1,4-bis[2-aminophenylthio] butadiene (U0126), rabbit anti-p27^Kip1^, rabbit anti-ERK1/2, and rabbit anti-phospho-ERK1/2 were purchased from Cell Signaling (Danvers, MA, USA). Polymerase chain reaction (PCR) primers were obtained from Eurofins MWG Operon (Ebersberg, Germany) and FastStart PCR Master Mix and Transcriptor High Fidelity cDNA Synthesis kit were from Roche Diagnostics (Mannheim, Germany). Bovine serum albumin (BSA) was from Calbiochem (San Diego, CA, USA) and polyvinylidene difluoride membranes were purchased from Millipore (Madrid, Spain). Amersham cGMP enzyme immunoassay Biotrak System, enhanced chemifluorescence reagent, and anti-rabbit and anti-mouse alkaline phosphatase-conjugated antibodies were from GE Healthcare Life Sciences (Buckinghamshire, UK). Other reagents used in immunoblotting experiments were purchased from Bio-Rad (Hercules, CA, USA).

### 2.2. Animals

C57BL/6J mice were obtained from Charles River (Barcelona, Spain) and kept in our animal facilities with food and water *ad libitum* in a 12-hour dark : light cycle. All experiments were performed in accordance with Institutional and European Guidelines (86/609/EEC) for the care and use of laboratory animals.

### 2.3. Subventricular Zone Cell Cultures

Neural stem cell cultures were obtained from the SVZ of 0–3 postnatal day C57BL/6J mice as previously described [30]. The fragments of SVZ were digested in 0.025% trypsin/0.265 mM EDTA, for 15–20 minutes at 37°C, and then mechanically dissociated. The cells were resuspended in a 37°C D-MEM/F-12 medium with 2 mM GlutaMAX-I, supplemented with 1% B27, 1% antibiotic (10,000 units/mL penicillin, 10 mg/mL streptomycin), 10 ng/mL EGF, and 5 ng/mL bFGF, and plated on uncoated flasks with filter cap, at a density of 100,000 cells/mL. The SVZ stem cells were grown as floating aggregates in a 95% air/5% CO_2_ humidified atmosphere at 37°C, during 7 days. Then, the primary neurospheres were harvested, centrifuged, and mechanically dissociated as single cells. Cells were replated as above and allowed to grow as secondary neurospheres. 6-7 days later, the floating neurospheres were collected, dissociated, and plated for 2-3 days on poly-L-lysine-coated 12-well plates in the same medium as above. In the 24 h preceding the experimental treatments, the cells were kept with a similar medium but without growth factors in order to ensure that the results observed following cell treatments were not due to the effect of EGF or bFGF.

### 2.4. Experimental Treatments

For cell proliferation analysis, the times of cell treatment with PDE5 inhibitors were chosen based on the times used previously to evaluate cell proliferation in SVZ-derived NSC using a NO-donor [9]. Thus, the cells were exposed for 6 h and 24 h to PDE5 inhibitors or stimulated for 24 h with the sGC activator, YC-1, or the cGMP analogue, 8-Br-cGMP (20 *μ*M). To analyze the phosphorylation of ERK1/2 and the levels of p27^Kip1^, different times were tested and SVZ-derived NSC were treated for 2 h in the following experiments, or as indicated in the figure legends. In both experiments, the stimuli applied were as follows: PDE inhibitors T0156 (1 *μ*M), sildenafil (1 *μ*M), and zaprinast (10 *μ*M) or sGC activator YC-1 (20 *μ*M) were incubated alone or together with the MEK1/2 inhibitor U0126 (1 *μ*M), the PKG inhibitor KT5823 (1 *μ*M), or the sGC inhibitor ODQ (50 *μ*M). U0126, KT5823, and ODQ were applied 30 min before exposure to PDE5 inhibitors and YC-1 and kept throughout the incubation period. For determination of cGMP levels, cells were treated with T0156 (1 *μ*M), sildenafil (1 *μ*M), or zaprinast (10 *μ*M) for 6 h. All the experiments were performed together with the respective controls (untreated cells).

### 2.5. Analysis of Cell Proliferation by Flow Cytometry

SVZ-derived neural stem cell proliferation was assessed by incorporation of ethynyl-2′-deoxyuridine (EdU) using the Click-iT EdU Alexa Fluor 488 Flow Cytometry Assay kit, as previously described [31]. 10 *μ*M EdU was added to the SVZ cultures for 6 h or 4 h before fixation, for the cells treated for 6 h or 24 h, respectively. For fixation, cells were washed with sterile 0.01 M PBS and then sterile StemPro Accutase cell dissociation reagent was added for 20 min, at 37°C. Cell fixation was performed in 70% ethanol overnight, at 4°C, as described previously [5, 31]. Detection of EdU incorporation was based on click chemistry, a copper catalyzed covalent reaction between an azide (conjugated with the Alexa Fluor 488 fluorophore) and an alkyne (EdU). Briefly, cells incubated for 30 minutes with the azide conjugate and copper sulphate, at room temperature, protected from light. Next, the cells were incubated with ribonuclease A and with the nuclear dye 7-actinomycin D (7-AAD), widely used as a cell viability and cell cycle marker, for 30 min, EdU incorporation and 7-AAD signal were detected on a BD FACScalibur Flow Cytometer, using the Cellquest Pro software, version 0.3.efab (Becton Dickinson, San Jose, CA, USA). Thirty thousand events were acquired per each experiment in the region of interest (including apoptotic cells, and G0/G1, S and G2/M cell cycle phases). A minimum of 4 independent experiments was analyzed for each condition. Data were analyzed using the WinMDI2.9 software and are presented as means ± SEM of the number of nonapoptotic cells that incorporated EdU (% of control), as previously described [31].

### 2.6. Western Blot Analysis

Cells were scraped and lysed in 50 mM Tris-HCl, 0.15 M NaCl, 1 mM EDTA, 1% Igepal, and 10% glycerol, supplemented with 200 *μ*M phenylmethylsulfonyl fluoride, 1 *μ*g/mL chymostatin, 1 *μ*g/mL leupeptin, 1 *μ*g/mL antipain, 1 *μ*g/mL pepstatin A, 1 mM sodium orthovanadate, 1 *μ*M dithiothreitol, and 5 mM NaF, pH 7.5, 4°C. Protein concentration was determined by the BCA method (BCA Protein Assay kit, Pierce, Rockford, IL, USA), according to manufacturer's instructions. 6x concentrated sample buffer was added and samples were denatured at 95°C for 5 min. Next, samples were used for protein separation by electrophoresis in sodium dodecyl sulfate (SDS)-polyacrylamide gels using MiniPROTEAN 3 systems (Bio-Rad Laboratories). Equal amounts of protein were separated by electrophoresis, and, then, electrophoretically transferred to the activated polyvinylidene difluoride membranes. Membranes were blocked by 1 h incubation, at room temperature, with Tris-buffered saline (137 mM NaCl, 20 mM Tris-HCl, pH 7.6) containing 0.1% Tween-20 (TBS-T) and 3% BSA. Incubations with the primary antibodies (rabbit phospho-ERK1/2 or rabbit anti-p27^kip1^, 1 : 1,000) in TBS-T containing 1% blocking solution were performed overnight, at 4°C. Next, the incubation with the appropriated alkaline phosphatase-linked secondary antibodies (anti-rabbit or anti-mouse, 1 : 20,000 in TBS-T containing 1% blocking solution) was performed at room temperature, during 1 h. After extensive washing in TBS-T followed by the incubation of the membranes with the enhanced chemifluorescence reagent, immunoreactive bands were visualized in the VersaDoc 3000 imaging system (Bio-Rad, Hercules, CA, USA). Data were analyzed with the Quantity One software version 4.6.9 (Bio-Rad Laboratories). Protein control loadings were either performed after membranes reactivation (5–10 s in 100% methanol and 20 min in TBS-T) using primary antibodies against rabbit ERK1/2 (1 : 1,000) or mouse *α*-tubulin (1 : 10,000).

### 2.7. Determination of cGMP Levels

cGMP levels in cultured SVZ-derived stem cells were determined after exposure to drugs for 6 h, using a cGMP Enzyme immunoassay Biotrak System. This experimental assay is based on competition between unlabelled cGMP from cell lysates and a fixed quantity of peroxidase-labelled cGMP, for a limited number of binding sites on a cGMP specific antibody coated on a 96-well plate. Cell lysis and cGMP measurement were performed according to manufacturer's instructions and as previously described [32]. Optical density was read at 450 nm. All the experiments were carried out in duplicate. The results are expressed as femtomoles per million of cells.

### 2.8. Data Analysis

Data are expressed as means ± SEM. Statistical significance was determined by using two-tailed *t*-tests or one-factor analysis of variance (ANOVA) followed by Bonferroni's or Dunnett's post-tests, as appropriate and indicated in the figure legends. Differences were considered significant when *P* < 0.05. The software used was GraphPad Prism 5.0 (GraphPad Software, La Jolla, CA, USA).

## 3. Results

### 3.1. Characterization of SVZ Primary Cultures

To investigate the expression of phosphodiesterases in SVZ-derived neural stem cell cultures, we assessed the presence of the cGMP-specific PDE5A, PDE9A, and PDE6C by PCR. Despite PDE6 presence is only described in retina photoreceptors [33], we also studied its expression in SVZ-derived NSC cultures since some of the inhibitors for PDE5 also act on PDE6. The expression of several PDEs normally found in the brain was analyzed in SVZ cultures by reverse-transcription PCR. We studied the presence of the cGMP-specific isoforms, PDE5A, PDE6C, and PDE9A. We also assessed the expression of PDE1 (isoforms PDE1A, 1B and 1C), and PDE10A, which hydrolyze both cAMP and cGMP, as well as the cAMP-specific PDE7 enzyme isoforms, PDE7A, and PDE7B, whose presence in the brain is well documented [34–36]. We observed the presence of PDE1, PDE5, PDE7 and PDE9 in SVZ cultures, while PDE10 is absent (see Supplementary Figure 1 in Supplementary materials available online at: http://dx.doi.org/10.1155/2014/878397). Moreover, expression of PDE6 catalytic subunit was not detected in SVZ-derived NSC cultures, but it was detected in the retina.

### 3.2. Inhibition of PDE5 Increases Cell Proliferation

**Figure 1:**
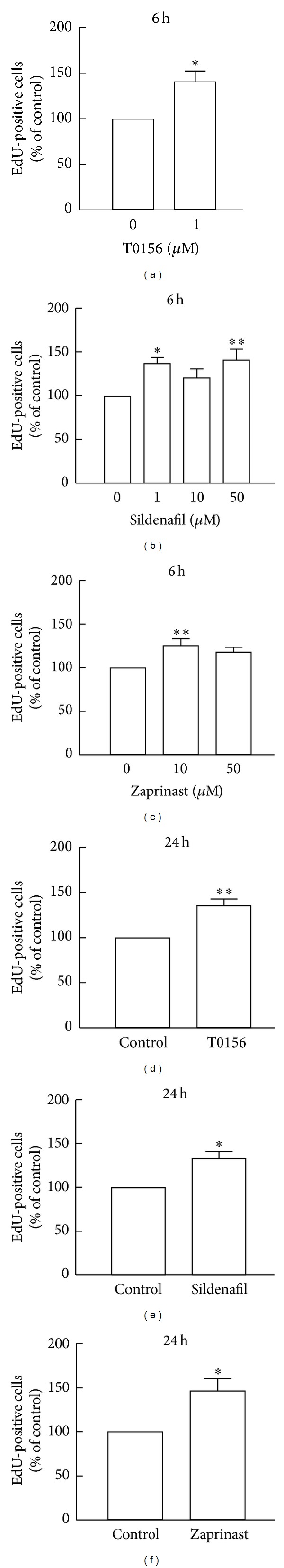
The PDE5 inhibitors T0156, sildenafil, and zaprinast stimulated the proliferation of SVZ neural stem cells following a 6 h or 24 h exposure. Cells were treated with 1 *μ*M T0156 (a), 1 *μ*M, 10 *μ*M and 50 *μ*M sildenafil (b), or 10 *μ*M and 50 *μ*M zaprinast (c) for 6 h, and with 1 *μ*M T0156 (d), 1 *μ*M sildenafil (e), or 10 *μ*M zaprinast (f) for 24 h. The incorporation of EdU was assessed by flow cytometry. Data are expressed as means ± SEM of at least 4 independent experiments. (a), (d), (e), and (f) two-tailed *t*-test, *P < 0.05 and **P < 0.01, significantly different from control and, (b) and (c) one-way ANOVA (Dunnett's post-test), *P < 0.05 and **P < 0.01, significantly different from control.

Neural stem cell proliferation was evaluated by assessing the incorporation of EdU by flow cytometry, following exposure to PDE5 inhibitors for 6 h. All PDE5 inhibitors increased the proliferation of SVZ-derived NSC. Treatment with T0156 (1 *μ*M [37]) significantly increased the incorporation of EdU (140.7 ± 11.9% of the control, *P* < 0.05; Figure 1(a)), when compared to control cultures (no treatment). Both sildenafil and zaprinast increased EdU incorporation, in comparison to untreated cultures, as follows: sildenafil (1 *μ*M, 137.4 ± 7.0%, *P* < 0.05; 10 *μ*M, 120.8 ± 10.4%, *P* > 0.05; 50 *μ*M, 141.3 ± 12.6%, *P* < 0.01; Figure 1(b)) and zaprinast (10 *μ*M, 125.6 ± 7.8%, *P* < 0.01; 50 *μ*M, 118.1 ± 5.6%, *P* > 0.05; Figure 1(c)). According to these data, 1 *μ*M sildenafil and 10 *μ*M zaprinast were selected for all the subsequent experiments.

In order to investigate whether this effect of PDE5 inhibitors on SVZ-derived NSC proliferation was maintained for longer times of exposure, cells were treated for 24 h with T0156, sildenafil, or zaprinast, and EdU incorporation was assessed by flow cytometry. The data presented in Figure 1 shows that, following 24 h of treatment, T0156 (1 *μ*M; 135.6 ± 7.3% of the control, *P* < 0.01; Figure 1(d)), sildenafil (1 *μ*M; 133.2 ± 8.2%, *P* < 0.05; Figure 1(e)) or zaprinast (10 *μ*M; 146.5 ± 13.9%, *P* < 0.05; Figure 1(f)) increased SVZ-derived NSC proliferation, in comparison to untreated cultures. The incorporation of EdU was further assessed by microscopy analysis. Likewise, following 24 h of treatment, T0156 (1 *μ*M; 182.4 ± 11.8% of the control, *P* < 0.001; Supplementary Figures 2(a) and 2(b)), sildenafil (1 *μ*M; 157.6 ± 8.9%, *P* < 0.01; Supplementary Figures 2(a) and 2(b)), or zaprinast (10 *μ*M; 142.1% ± 14.1%, *P* < 0.05; Supplementary Figures 2(a) and 2(b)) increased SVZ-derived NSC proliferation, in comparison to untreated cultures (100% corresponding to 2.7 ± 0.4% of the total of live cells). Moreover, treatment with these concentrations of PDE5 inhibitors did not have a cytotoxic effect on SVZ cultures (Supplementary Table 2), as evaluated by detection of the nuclear dye signal, 7-AAD, by flow cytometry.

### 3.3. PDE5 Inhibition Increases cGMP Levels

To investigate whether PDE5 inhibition resulted, in fact, in increased cGMP levels, the levels of cGMP were measured following exposure to different PDE5 inhibitors. As shown in Table 1, we observed that cGMP levels were significantly increased following exposure to T0156 (29.7 ± 4.2 fmol/10^6^ cells, *P* < 0.05), sildenafil (28.8 ± 5.1 fmol/10^6^ cells, *P* < 0.05), or zaprinast (33.1 ± 6.1 fmol/10^6^ cells, *P* < 0.01) as compared to cGMP levels measured in untreated cultures (7.4 ± 0.9 fmol/10^6^ cells).

### 3.4. PDE5 Inhibition Stimulates Cell Proliferation via the ERK/MAPK Pathway

**Figure 2:**
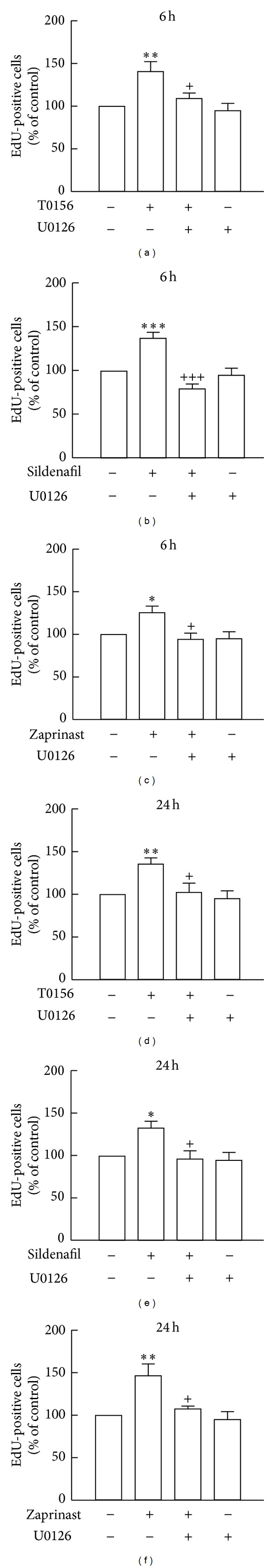
Inhibition of the ERK1/2 pathway prevented proliferation of SVZ cells stimulated by PDE5 inhibitors. Cell proliferation following treatment with 1 *μ*M U0126 and 1 *μ*M T0156 (a, d), 1 *μ*M sildenafil (b, e) or 10 *μ*M zaprinast (c, f) was assessed by incorporation of EdU and evaluated by flow cytometry following 6 h and 24 h of treatment. Data are expressed as means ± SEM of at least 4 independent experiments. One-way ANOVA (Bonferroni's post-test), *P < 0.05, **P < 0.01, and ***P < 0.001, significantly different from control and ^+^ *P* < 0.05, and ^+++^ *P* < 0.001, significantly different from the PDE5 inhibitor.

To evaluate the involvement of the ERK/MAPK pathway on cell proliferation stimulated by PDE5 inhibition, SVZ-derived NSC cultures were treated with the MEK1/2 inhibitor, U0126. Exposure to U0126 for 6 h prevented the increased EdU incorporation triggered by T0156 (109.1 ± 6.6% of the control, *P* < 0.05; Figure 2(a)), sildenafil (79.5 ± 5.8%, *P* < 0.001; Figure 2(b)), or zaprinast (94.3 ± 7.4%, *P* < 0.05; Figure 2(c)), keeping cell proliferation similar to control cultures, in comparison to T0156 (6 h, 140.7 ± 11.9%, *P* < 0.01), sildenafil (6 h, 137.4 ± 7.0%, *P* < 0.001), or zaprinast (6 h, 125.6 ± 7.8%, *P* < 0.05) alone, respectively. Similar results were obtained following 24 h of treatment. In the presence of U0126, the increase in the incorporation of EdU by T0156 (102.5 ± 10.7% of the control, *P* < 0.05; Figure 2(d)), sildenafil (96.7 ± 9.6%, *P* < 0.05; Figure 2(e)), or zaprinast (107.6% ± 3.4%, *P* < 0.05; Figure 2(f)) was prevented, comparatively to T0156 (24 h, 135.6 ± 7.3%, *P* < 0.01), sildenafil (24 h, 133.2 ± 8.2%, *P* < 0.05) or zaprinast (24 h, 146.5 ± 13.9%, *P* < 0.01) alone, respectively.

### 3.5. PDE5 Inhibition Increases ERK1/2 Phosphorylation

Exposure to NO triggers cell proliferation in SVZ-derived NSC cultures by activation of ERK1/2 signaling, which can be monitored by evaluating the phosphorylation of ERK1/2 and the concomitant decrease in the levels of cyclin-dependent kinase inhibitor 1, p27^Kip1^ [5]. p27^Kip1^ is regulated by p90 RSK and, once phosphorylated, is translocated to the cytosol, followed by ubiquitination and degradation by the proteosome, allowing progression to S-phase [38].

In order to choose the best time to study the effect of PDE5 inhibition, or sGC activation, on the levels of phospho-ERK1/2 and p27^Kip1^, cultures were exposed to T0156 (1 *μ*M; Supplementary Figure 3(a)), sildenafil (1 *μ*M; Supplementary Figure 3(b)), zaprinast (10 *μ*M; Supplementary Figure 3(c)), or YC-1 (20 *μ*M; Supplementary Figure 3(d)) for 30 min, 1 h, or 2 h. Treatment with T0156, zaprinast, and YC-1 increased ERK1/2 phosphorylation following 2 h of treatment. Furthermore, the cellular levels of p27^Kip1^ appear to be decreased, mainly following a 2 h treatment with T0156. According to these results, an exposure of 2 h to T0156, sildenafil, zaprinast, or YC-1 was used in these experiments.

**Figure 3:**
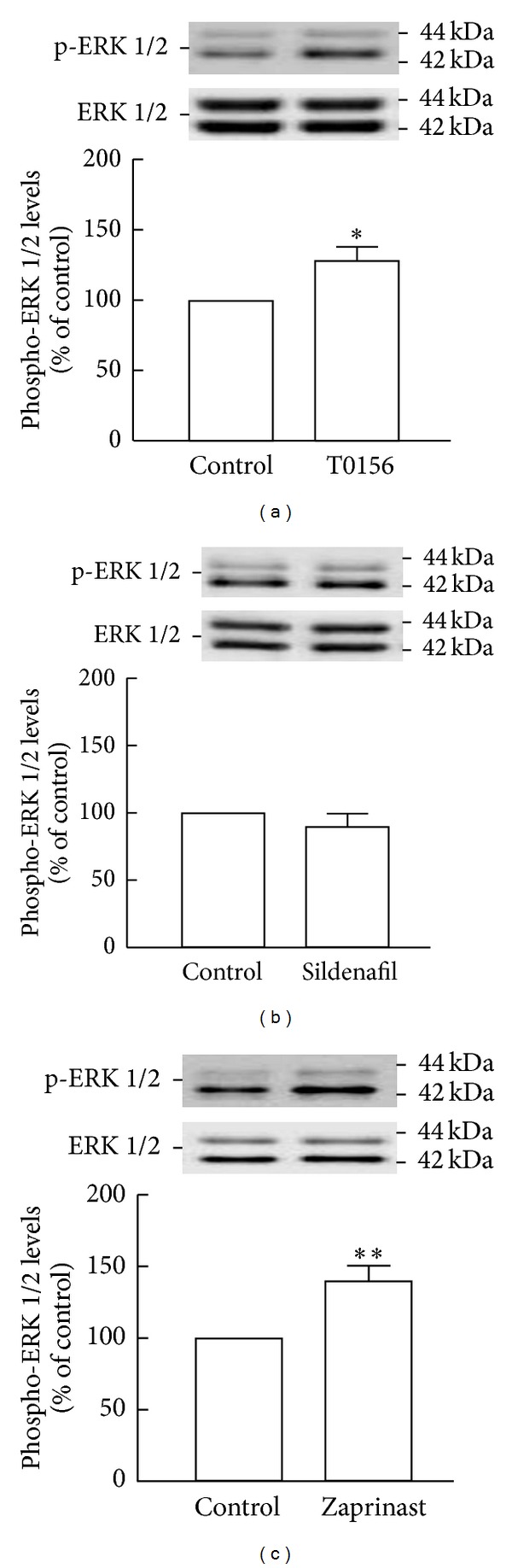
T0156 or zaprinast, but not sildenafil, increased ERK1/2 phosphorylation following 2 h of treatment. Phospho-ERK1/2 levels following treatment with 1 *μ*M T0156 (a), 1 *μ*M sildenafil (b), and 10 *μ*M zaprinast (c) for 2 h were assessed by Western blot. Representative images are shown. Data are expressed as means ± SEM of at least 5 independent experiments. Two-tailed *t*-test, *P < 0.05 and **P < 0.01, significantly different from control.

Treatment with T0156 (1 *μ*M; 128.5 ± 10.0% of the control, *P* < 0.05; Figure 3(a)) or zaprinast (10 *μ*M; 140.0 ± 11.0%, *P* < 0.01; Figure 3(c)) significantly increased ERK1/2 phosphorylation in comparison to control cultures. However, treatment with sildenafil (1 *μ*M; 89.9 ± 9.8%, *P* > 0.05; Figure 3(b)) did not increase the levels of phospho-ERK1/2 in relation to untreated cultures. Since a short period of exposure to sildenafil (1 *μ*M, for 2 h) did not affect phospho-ERK1/2 levels, we decided to test a longer time of exposure (6 h). Interestingly, we did not observe any remarkable differences in phospho-ERK1/2 (106.8 ± 8.5%, *P* > 0.05; Supplementary Figure 4(a)) following exposure to sildenafil for 6 h, when compared to control cultures.

### 3.6. PDE5 Inhibition Enhances Cell Proliferation via sGC/PKG Pathway

**Figure 4:**
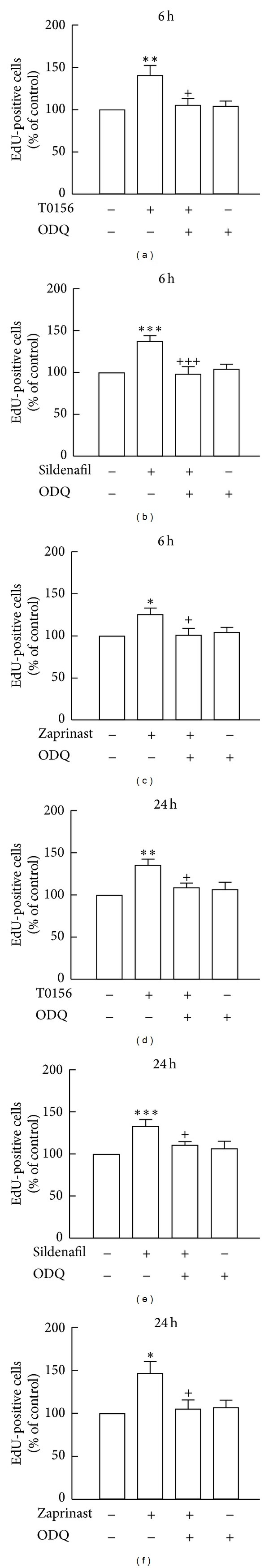
Inhibition of sGC prevented the proliferation of SVZ cells stimulated by PDE5 inhibitors. Cell proliferation following treatment with 50 *μ*M ODQ and 1 *μ*M T0156 (a, d), 1 *μ*M sildenafil (b, e), or 10 *μ*M zaprinast (c, f) was assessed by incorporation of EdU and evaluated by flow cytometry, following 6 h or 24 h treatment. Data are expressed as means ± SEM of at least 5 independent experiments. One-way ANOVA (Bonferroni's post-test), *P < 0.05, **P < 0.01 and ***P < 0.001, significantly different from control and ^+^ *P* < 0.05 and ^+++^ *P* < 0.001 significantly different from the PDE5 inhibitor.

To analyze whether the sGC/PKG pathway is involved in cell proliferation stimulated by PDE5 inhibition, SVZ-derived NSC cultures were exposed to the sGC inhibitor, ODQ, or the PKG inhibitor, KT5823. We found that ODQ prevented cell proliferation stimulated by T0156 (105.5 ± 7.9% of the control, *P* < 0.05; Figure 4(a)), sildenafil (98.3 ± 9.0%, *P* < 0.001; Figure 4(b)), and zaprinast (101.1 ± 8.0%, *P* < 0.05; Figure 4(c)), as compared to T0156 (140.7 ± 11.9%, *P* < 0.01), sildenafil (137.4 ± 7.0%, *P* < 0.001), or zaprinast (125.6 ± 7.8%, *P* < 0.05) alone, respectively. Moreover, following 24 h of treatment, ODQ is also effective in preventing the increase of EdU-positive cells by T0156 (109.1 ± 5.5%, *P* < 0.05; Figure 4(d)), sildenafil (110.8 ± 4.4%, *P* < 0.05, Figure 4(e)), and zaprinast (105.1 ± 10.8%, *P* < 0.05, Figure 4(f)), in comparison to T0156 (135.6 ± 7.3%, *P* < 0.01), sildenafil (24 h, 133.2 ± 8.2%, *P* < 0.001), or zaprinast (146.5 ± 13.9%, *P* < 0.05) alone, respectively.

**Figure 5:**
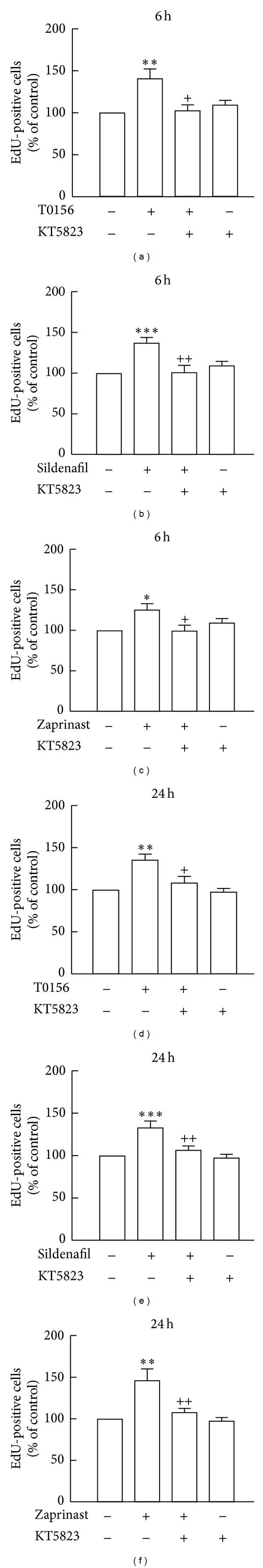
Inhibition of PKG prevented the proliferation of SVZ cells stimulated by PDE5 inhibitors. Cell proliferation following treatment with 1 *μ*M KT5823 and 1 *μ*M T0156 (a, d), 1 *μ*M sildenafil (b, e), or 10 *μ*M zaprinast (c, f) was assessed by incorporation of EdU and evaluated by flow cytometry, following 6 h or 24 h of treatment. Data are expressed as means ± SEM of at least 4 independent experiments. One-way ANOVA (Bonferroni's post-test), *P < 0.05, **P < 0.01 and ***P < 0.001, significantly different from control and ^+^ *P* < 0.05 and ^++^ *P* < 0.01 significantly different from the PDE5 inhibitor.

By inhibiting PKG with KT5823, following 6 h of treatment with PDE5 inhibitors, we observed a prevention of the increase in cell proliferation induced by T0156 (102.6 ± 7.1% of the control, *P* < 0.05; Figure 5(a)), sildenafil (101.1 ± 9.0%, *P* < 0.01; Figure 5(b)), and zaprinast (99.5 ± 7.4%, *P* < 0.05; Figure 5(c)), and cell proliferation became similar to basal levels, whereas the response to T0156 (6 h, 140.7 ± 11.9%, *P* < 0.01), sildenafil (6 h, 137.4 ± 7.0%, *P* < 0.001) or zaprinast (6 h, 125.6 ± 7.8%, *P* < 0.05) alone, respectively, was maintained. Similarly, 24 h of treatment with KT5823 prevented the increase in cell proliferation induced by T0156 (108.5 ± 7.9%, *P* < 0.05; Figure 5(d)), sildenafil (106.8 ± 5.3%, *P* < 0.01; Figure 5(e)), and zaprinast (108.1 ± 4.9%, *P* < 0.01; Figure 5(f)), in comparison to T0156 (24 h, 135.6 ± 7.3%, *P* < 0.01), sildenafil (24 h, 133.2 ± 8.2%, *P* < 0.001), or zaprinast (24 h, 146.5 ± 13.9%, *P* < 0.01) alone, respectively.

### 3.7. PKG Is Involved in ERK1/2 Phosphorylation Induced by PDE5 Inhibition

**Figure 6:**
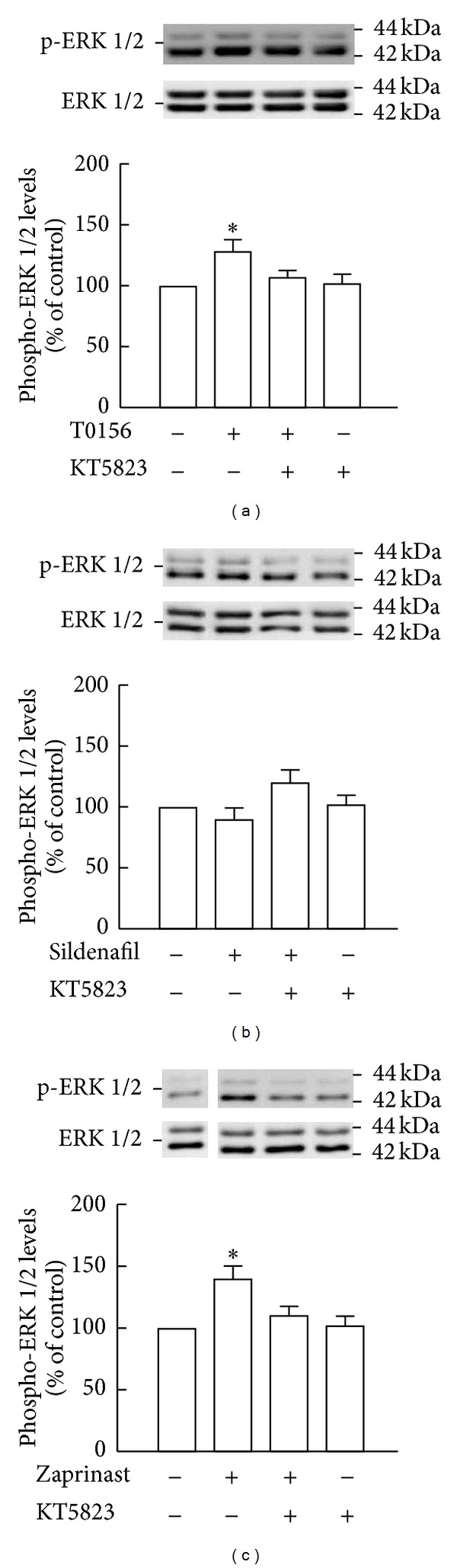
Inhibition of PKG prevented the phosphorylation of ERK1/2 by treatment with T0156 or zaprinast. Levels of phospho-ERK1/2 following treatment with 1 *μ*M KT5823 and 1 *μ*M T0156 (a), 1 *μ*M sildenafil (b), and 10 *μ*M zaprinast (c) for 2 h were assessed by Western blot. Representative images are shown. Data are expressed as means ± SEM of at least 4 independent experiments. One-way ANOVA (Bonferroni's post-test), *P < 0.05, significantly different from control.

In order to evaluate whether inhibiting PKG with KT5823 would antagonize the effect of PDE5 inhibition by T0156 or zaprinast on ERK1/2 phosphorylation, samples obtained from SVZ-derived NSC cultures were analyzed by Western blot, following 2 h of treatment. Treatment with KT5823 suppressed the increase in phospho-ERK1/2 levels induced by T0156 (107.2 ± 6.2% of the control, *P* > 0.05; Figure 6(a)) or zaprinast (110.5 ± 7.9%, *P* > 0.05; Figure 6(c)) in comparison to T0156 (128.5 ± 10.0%, *P* < 0.05) and zaprinast (140.0 ± 11.0%, *P* < 0.05) alone, respectively. On the contrary, as sildenafil did not stimulate ERK1/2 phosphorylation (Figure 3(b)), treatment with KT5823 did not change phospho-ERK1/2 levels (120.2 ± 11.0%, *P* > 0.05; Figure 6(b)) when compared to sildenafil alone (89.9 ± 9.8%, *P* > 0.05).

### 3.8. T0156 Treatment Decreases p27^Kip1^ Levels

**Figure 7:**
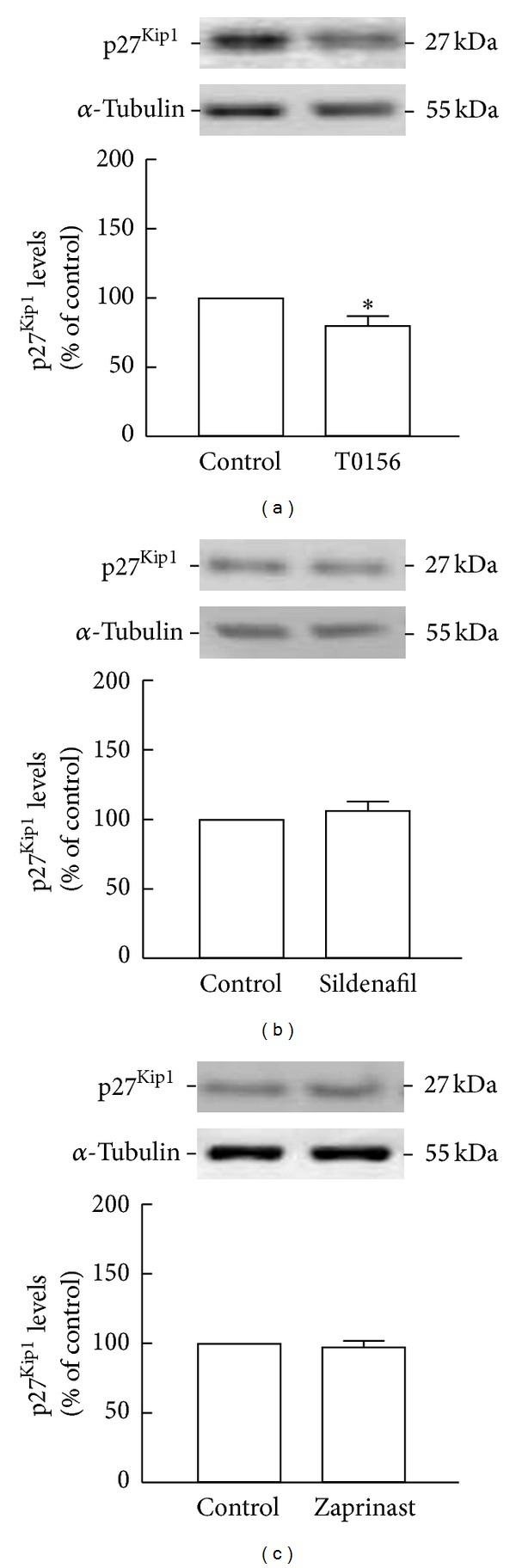
Treatment with T0156 decreased p27^Kip1^ levels. p27^Kip1^ levels following treatment 1 *μ*M T0156 (a), 1 *μ*M sildenafil (b), or 10 *μ*M zaprinast (c) for 2 h were assessed by Western blot. Representative images are shown. Data are expressed as means ± SEM of at least 6 independent experiments. Two-tailed *t*-test, *P < 0.05, significantly different from control.

Next, we evaluated whether p27^Kip1^ levels were altered following 2 h of exposure to T0156, sildenafil, or zaprinast, by Western blot. We observed that, following treatment with T0156, p27^Kip1^ levels significantly decreased (80.0 ± 7.2% of the control, *P* < 0.05; Figure 7(a)) in comparison to the levels found in untreated cultures. On the contrary, treatment with sildenafil (106.4 ± 6.7%, *P* > 0.05; Figure 7(b)) or zaprinast (97.4 ± 4.8%, *P* > 0.05; Figure 7(c)) did not change p27^Kip1^ levels in comparison to control cultures. Moreover, similar to what was observe for ERK1/2 phosphorylation, following a longer exposure to sildenafil, for 6 h, we did not observe any remarkable differences in the levels of p27^Kip1^ (91.7 ± 8.9%, *P* > 0.05; Supplementary Figure 4(b)), when compared to control.

**Figure 8:**
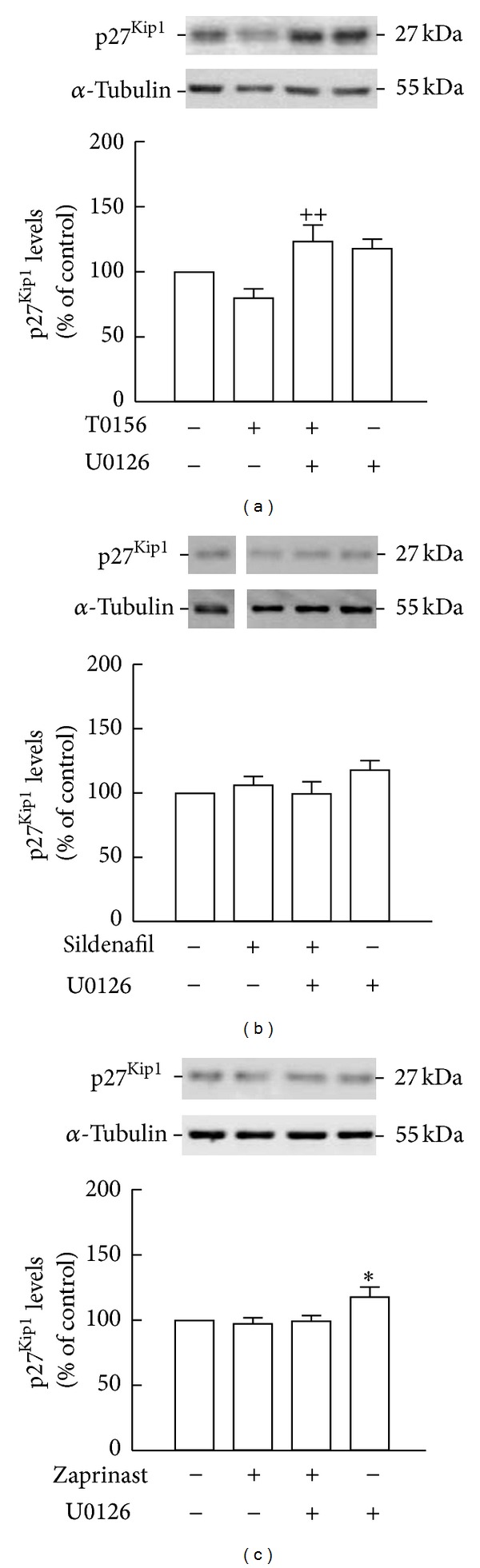
Inhibition of MAPK pathway prevented the decrease of p27^Kip1^ levels mediated by T0156. p27^Kip1^ levels following treatment with 1 *μ*M U0126 and 1 *μ*M T0156 (a), 1 *μ*M sildenafil (b), or 10 *μ*M zaprinast (c) for 2 h were assessed by Western blot. Representative images are shown. Data are expressed as means ± SEM of at least 5 independent experiments. One-way ANOVA (Bonferroni's post-test), *P < 0.05, significantly different from control and ^++^ *P* < 0.01, significantly different from T0156.

In order to test whether this effect can be reversed by inhibiting the ERK1/2 pathway, NSC cultures were treated with U0126 for 2 h. By Western blotting, we observed that U0126 alone increased p27^Kip1^ levels above the baseline (118.0 ± 7.5%; Figure 8). Furthermore, the levels of p27^Kip1^ were significantly increased following treatment with U0126 plus T0156 (123.5 ± 13.0% of the control, *P* < 0.01; Figure 8(a)), comparatively to T0156 (80.0 ± 7.2%, *P* > 0.05) alone. On the other hand, U0126 did not change the levels of p27^Kip1^ in samples treated with sildenafil (99.6 ± 9.6%, *P* > 0.05; Figure 8(b)) or zaprinast (99.3 ± 4.6%, *P* > 0.05; Figure 8(c)) as compared to sildenafil (106.4 ± 6.7%, *P* > 0.05) or zaprinast (97.4 ± 4.8%, *P* > 0.05) alone, respectively.

**Figure 9:**
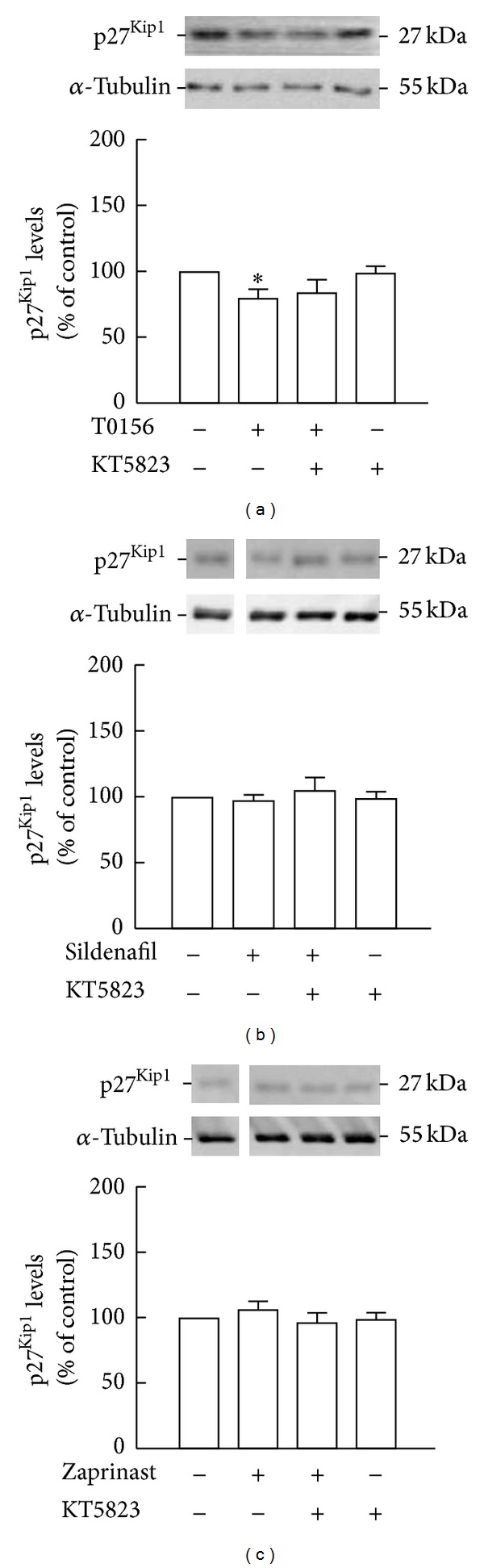
Inhibition of PKG did not prevent the decrease of p27^Kip1^ levels by T0156. p27^Kip1^ levels following treatment with 1 *μ*M KT5823 and 1 *μ*M T0156 (a), 1 *μ*M sildenafil (b), or 10 *μ*M zaprinast (c) for 2 h were assessed by Western blot. Representative images are shown. Data are expressed as means ± SEM of at least 4 independent experiments. One-way ANOVA (Bonferroni's post-test), *P < 0.05, significantly different from control.

We also observed that inhibition of PKG did not appear to affect the levels of p27^Kip1^ following treatment with T0156 (84.1 ± 10.2%, *P* > 0.05; Figure 9(a)), sildenafil (96.3 ± 7.8%, *P* > 0.05; Figure 9(b)), or zaprinast (105.1 ± 10.0%, *P* > 0.05; Figure 9(c)), in comparison to T0156 (80.0 ± 7.2%, *P* < 0.05), sildenafil (106.4 ± 6.7%, *P* > 0.05), or zaprinast (97.4 ± 4.8%, *P* > 0.05) alone, respectively.

### 3.9. YC-1 Stimulates Cell Proliferation via the sGC/PKG and ERK/MAPK Pathways

**Figure 10:**
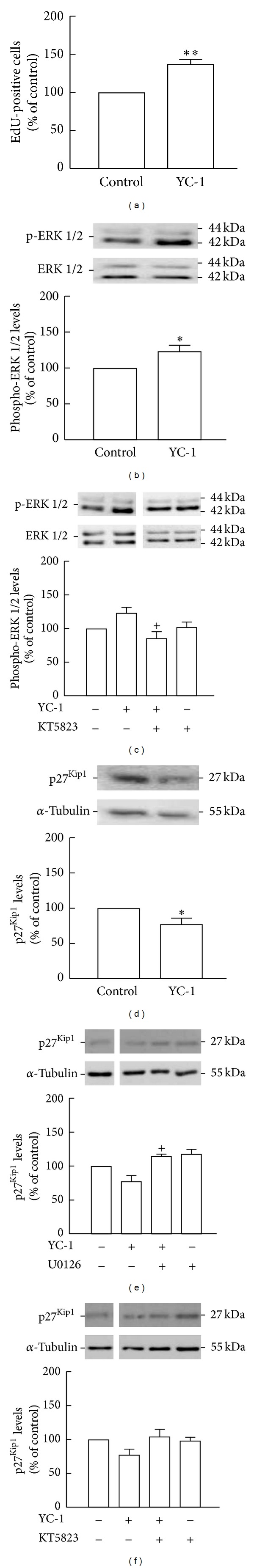
Activation of sGC stimulates proliferation of NSC, increases ERK1/2 phosphorylation, and decreases p27^Kip1^ levels. Cell proliferation following treatment with 20 *μ*M YC-1 (a) for 24 h was assessed by the incorporation of EdU and analyzed by flow cytometry. Levels of phospho-ERK1/2 following treatment with 20 *μ*M YC-1 (b) or YC-1 plus 1 *μ*M KT5823 (c) and p27^Kip1^ levels following treatment with YC-1 (d) or YC-1 plus 1 *μ*M U0126 (e) or KT5823 (f) for 2 h were assessed by Western blot. Representative images are shown. Data are expressed as means ± SEM of at least 4 independent experiments. (a), (b), and (d) two-tailed *t*-test, *P < 0.05 and **P < 0.01, significantly different from control and (c), (e), and (f) one-way ANOVA (Bonferroni's post-test), ^+^ *P* < 0.05, significantly different from YC-1.

To evaluate the effect on the proliferation of NSC of increasing the levels of cGMP, by direct activation of sGC, SVZ-derived NSC cultures were treated with the sGC activator, YC-1 (20 *μ*M). EdU incorporation was assessed by flow cytometry and microscopy analysis. Exposure to YC-1, for 24 h, increased the incorporation of EdU by NSC (137.1 ± 6.8% of the control, *P* < 0.01; Figure 10(a)), when compared to untreated cultures, as evaluated by flow cytometry. Similarly, evaluating EdU labeling by microscopy, the increase in cell proliferation following 24 h of treatment with YC-1 was also observed (145.8 ± 12.6%, *P* < 0.05; Supplementary Figures 2(a) and 2(c)), in comparison to untreated cultures. Treatment with the same concentration of YC-1 for 6 h did not alter the percentage of EdU-positive cells in comparison to control cultures (data not shown). Furthermore, treatment with the cGMP-analogue, 8-Br-cGMP, also increased proliferation of NSC (20 *μ*M; 145.9 ± 11.9%, *P* < 0.05; Supplementary Figures 2(a) and 2(d)), when compared to control cultures.

In order to evaluate whether direct activation of sGC would also affect phosphorylation of ERK1/2 and p27^Kip1^ levels, samples obtained from SVZ-derived NSC cultures were treated with the sGC activator. ERK1/2 phosphorylation and p27^Kip1^ levels were analyzed by Western blot, following 2 h of treatment. In fact, treatment with YC-1 significantly increased ERK1/2 phosphorylation (123.3 ± 8.9%, *P* < 0.05; Figure 10(b)), in comparison to control cultures. Moreover, inhibition of PKG with KT5823 prevented the increase in phospho-ERK1/2 levels due to exposure to YC-1 (85.6 ± 10.4%, *P* < 0.05; Figure 10(c)) when compared to YC-1 alone (123.3 ± 8.9%, *P* > 0.05).

Furthermore, similar to T0156, the levels of p27^Kip1^ were significantly decreased following treatment with YC-1 (77.5 ± 9.0%, *P* < 0.05, Figure 10(d)), when compared to untreated cultures. This effect can be reversed by inhibiting the ERK1/2 pathway, with U0126, as the levels of p27^Kip1^ were significantly increased following treatment with U0126 plus YC-1 (114.9 ± 3.4%, *P* < 0.05; Figure 10(e)), comparatively to YC-1 (77.5 ± 9.0%, *P* > 0.05) alone. Inhibition of PKG also appeared to prevent the decrease in p27^Kip1^ levels induced by YC-1, as treatment with KT5823 and YC-1 tended to raise p27^Kip1^ levels close to basal (104.5 ± 11.3%, *P* > 0.05; Figure 10(f)) when compared to YC-1 alone (77.5 ± 9.0%, *P* > 0.05).

## 4. Discussion

In this work, we show that proliferation of NSC is stimulated by cGMP, since inhibition of cGMP hydrolysis by PDE5 enhances the proliferation of NSC isolated from SVZ. Moreover, direct activation of sGC by the activator YC-1 has a similar effect in increasing NSC proliferation following 24 h of treatment. We also show that ERK1/2 is activated following treatment with PDE5 inhibitors (except sildenafil), suggesting the involvement of this pathway in cell proliferation mediated by PDE5 inhibition. Taken together, our data point out to the activation by PDE5 inhibition in SVZ cells of two pathways: the sGC/PKG and the ERK/MAPK pathways, the same pathways responsible for the proliferative effect of NO [9].

Using inhibitors with different selectivity for PDE5 we show that inhibition of PDE5 either with T0156, sildenafil, or zaprinast enhances the proliferation of NSC, the first step of neurogenesis. This is the first study comparing the proliferative potential of different PDE5 inhibitors on NSC. Several studies report the proneurogenic effect of sildenafil on SVZ cultures [39] as in animal models of brain injury [24–26], but the effect of sildenafil on neurogenesis was never compared to other PDE5 inhibitors. We show a significant increase in the levels of cGMP in NSC treated with PDE5 inhibitors, which is responsible for the increase in cell proliferation. Moreover, direct activation of sGC with YC-1, and, thus, increasing in cGMP production, also enhances NSC proliferation upon 24 h of treatment. Likewise, a cGMP analogue, 8-Br-cGMP, also enhances SVZ cell proliferation, as shown in this work and previously by our group [9].

Here, we show that, inhibiting the MAPK pathway, the proliferative effect of PDE5 inhibitors is abolished in both short- and long-term exposures, which is in agreement with the established contribution of ERK pathway to the proliferation of NSC [5]. In addition, cell proliferation induced by the PDE5 inhibitors was also prevented by inhibitors of sGC (ODQ) or PKG (KT5823), showing the involvement of sGC/PKG pathway. We have previously shown that both NO and cGMP analogues enhance SVZ proliferation by a mechanism that is dependent on activation of PKG, concomitantly with ERK activation [9]. The present study suggests that the same pathways involved in stimulating cell proliferation by the endogenous messenger (NO) are also being stimulated by blocking the hydrolysis of cGMP with PDE5 inhibitors.

T0156 and zaprinast were able to activate ERK1/2, an effect that is prevented by inhibition of PKG, suggesting that ERK1/2 activation is dependent on the cGMP effector kinase, PKG. Several other studies report the regulation of ERK1/2 activation by PKG, which is involved in multiple cell functions [9, 40–42]. Furthermore, treatment with T0156 decreases the levels of the cyclin-dependent kinase inhibitor 1, p27^Kip1^. p27^Kip1^ prevents progression from G1 to S-phase, being a key regulator of cell division of neural progenitor cells [43, 44]. The downregulation of p27^Kip1^ levels by T0156 is consistent with the increase in cell proliferation and ERK1/2 phosphorylation shown above. In agreement with this, we found that blockade of ERK/MAPK pathway, by inhibition of MAPK, is able to prevent this effect. Moreover, direct activation of sGC by YC-1 has a similar effect to that of T0156 in increasing phospho-ERK1/2 and decreasing p27^Kip1^ levels, similar to those previously described with NO or the cGMP analogue, 8-Br-cGMP [9]. However, although zaprinast increases ERK1/2 phosphorylation, it does not appear to affect the levels of p27^Kip1^. In fact, by the lack of selectivity of zaprinast for PDE5, we cannot exclude the possibility that this inhibitor could interact with other PDE and, thus, to activate other pathways, than the two studied here.

Interestingly, while sildenafil increased NSC proliferation, it neither increased ERK1/2 phosphorylation nor altered the levels of p27^Kip1^ for any of the times tested in this work. Even though ERK1/2 is not phosphorylated following treatment with sildenafil, we observed that the MAPK inhibitor, U0126, completely blocks sildenafil-enhanced cell proliferation, which suggests the involvement of other signaling pathways in the proliferative effect of this drug. A similar lack of effect of sildenafil on ERK1/2 phosphorylation has already been described by Wang and colleagues [39]. According to that study, sildenafil (300 nM) does not increase ERK1/2 phosphorylation following 1 h of treatment [39]. These authors suggest that the increase in cGMP levels via inhibition of PDE5 activity enhances neurogenesis in the SVZ through the PI3-K/Akt pathway [39]. Within this scenario, sildenafil appears to act in a proliferative pathway different from the one activated by NO, by cGMP analogues [9], and by the other PDE5 inhibitors used in this study. Thus, understanding the pathways activated by sildenafil is important in order to understand its possible value in the field of proneurogenic therapies.

Therapies involving the specific inhibition of PDE5 are being evaluated for the treatment of several neurological conditions. Regarding the possible enhancement of endogenous neurogenesis by this strategy, our study helps in better understanding the initial events that promote proliferation of NSC in the initial stages of neurogenesis by PDE5 inhibition.

## Supplementary Material

The expression of several PDEs normally found in the brain was analyzed in SVZ cultures by reverse-transcription PCR. We studied the presence of the cGMP-specific isoforms, PDE5A, PDE6C, and PDE9A. We also assessed the expression of PDE1 (isoforms PDE1A, 1B and 1C), and PDE10A, which hydrolyze both cAMP and cGMP, as well as the cAMP-specific PDE7 enzyme isoforms, PDE7A, and PDE7B, whose presence in the brain is well documented [34–36]. We observed the presence of PDE1, PDE5, PDE7 and PDE9 in SVZ cultures, while PDE10 is absent (see available online at: http://dx.doi.org//10.1155/2013/878397 Supplementary Figure 1). Moreover, expression of PDE6 catalytic subunit was not detected in SVZ-derived NSC cultures, but it was detected in the retina.

## Figures and Tables

**Table 1:** PDE5 inhibitors increased the levels of cGMP in SVZ cells.

Treatment	cGMP levels (fmol/10^6^ cells)
Control	7.4 ± 0.9
T0156	29.7 ± 4.2*
Sildenafil	28.8 ± 5.1*
Zaprinast	33.1 ± 6.1**

Cells were treated for 6 h with 1 *µ*M T0156, 1 *µ*M sildenafil, or 10 *µ*M zaprinast and then lysed to measure the levels of cGMP. Data are expressed as means ± SEM of 4 independent experiments. One-way ANOVA (Dunnett's post-test), *P < 0.05 and **P < 0.01, significantly different from control.
