# Supplementary material for: Stimulation of Neural Stem Cell Proliferation by Inhibition of Phosphodiesterase 5
Source: Stem Cells Int. 2014 Jan 12;2014:878397. doi: 10.1155/2014/878397 (PMC3914480; doi:10.1155/2014/878397)
Supplement: Supplementary file 1 — The expression of several PDEs normally found in the brain was analyzed in SVZ cultures by reverse-transcription PCR. We studied the presence of the cGMP-specific isoforms, PDE5A, PDE6C, and PDE9A. We also assessed the expression of PDE1 (isoforms PDE1A, 1B and 1C), and PDE10A, which hydrolyze both cAMP and cGMP, as well as the cAMP-specific PDE7 enzyme isoforms, PDE7A, and PDE7B, whose presence in the brain is well documented [34–36]. We observed the presence of PDE1, PDE5, PDE7 and PDE9 in SVZ cultures, while PDE10 is absent (see available online at: http://dx.doi.org//10.1155/2013/878397 Supplementary Figure 1). Moreover, expression of PDE6 catalytic subunit was not detected in SVZ-derived NSC cultures, but it was detected in the retina. [file 878397.f1.pdf]

Supplementary Methods

Analysis for PDE expression by reverse-transcription PCR

RNA was extracted from untreated SVZ-derived NSC by 2-Propanol/chloroform extraction procedure, using TRI Reagent. RNA extraction from a total mouse brain was used as positive control for all PDE tested, except for PDE6 (RNA from mouse retina was used). For both, RNA was reverse-transcribed by using random hexamer primers with the Transcriptor High Fidelity cDNA Synthesis kit, following the manufacturer's instructions. 100 ng of cDNA product was used in the PCR catalyzed by the DNA polymerase included in FastStart PCR Master Mix. The sequences of primers used are listed in Supplementary table **1**. An initial denaturation at 95 ºC for 4 min was performed on a thermal cycler followed by 40 cycles with 30 s of denaturation at 95 ºC, 30 s of annealing at 58 ºC (except for PDE10A, 53 ºC for 30 s) and 30 s of extension at 72 ºC, ending with a final extension of 7 min at 72 ºC. A portion of the PCR mixture was electrophoresed in a 2 % agarose gel in TAE 1x buffer, stained with ethidium bromide.

Analysis of cell proliferation by microscopy

Proliferation of SVZ-derived neural stem cells was assessed by incorporation of EdU using the Click-iT® EdU Alexa Fluor® 594 HCS Assay kit, as previously described [31]. Cells were treated for 24 h with PDE5 inhibitors T0156, sildenafil or zaprinast, with the sGC activator YC-1 or with the cGMP analogue 8-Br-cGMP. 10 µM EdU was added to the SVZ cultures for 4 h before fixation. Cells were fixed with 4% paraformaldehyde/4% sucrose, rinsed with 3

% BSA in PBS, and permeabilized with 0.5 % Triton X-100. Detection of EdU incorporation is based on click chemistry, a copper catalyzed covalent reaction between an azide (conjugated with the Alexa Fluor 594 fluorophore) and an alkyne (EdU). Cells were incubated with the azide conjugate and copper sulphate, following the manufacturer's instructions. Nuclei were labeled with Hoechst 33342. Images were acquired in an epifluorescence microscope (Axioimager Z2, Zeiss, Jena, Germany) with a Coolsnap HQ 2 camera (Photometrics, Arizona, USA). A minimum of 4 independent experiments was analyzed for each condition. Results are presented as means ± SEM of the percentage of live cells that incorporated EdU (% of control), as previously described [31].

Supplementary Results

Supplementary Table 1. Sequences of forward and reverse primers for mouse PDE1, 5, 6, 7, 9 and 10.

Gene	Forward/ Reverse	Sequence
PDE1A [1]	Forward	5’-GGGAAACTACAGTGCCATCTTC-3’
	Reverse	5’-CGCAATCCCTGAACTGTATGTA-3’
PDE1B [1]	Forward	5’-CTCATCCGACCAATGTCTGTAA-3’
	Reverse	5’-TACAAGAGAGGAGGAGGCAGTC-3’
PDE1C [1]	Forward	5’-CCTGAGCAGATCGAGAAAATCT-3’
	Reverse	5’-CCGTCTGTACATTCTTTCCACA-3’
PDE5A [2]	Forward	5’-AATACCACCCCTGGAGCACC-3’
	Reverse	5’-TTCAAGGGCTCGCCAAAAGC-3’
PDE6C	Forward	5’-ACGTAACCTCCGACCCGACCA-3’
	Reverse	5’-CATAGGCTGACTCTGCACCTCCCA-3’
PDE7A [3]	Forward	5’-AGTCTATTGCCAACATCCAGATTG-3’
	Reverse	5’-GAAAACCTGGCCCACTCTGTAA-3’
PDE7B [3]	Forward	5’-TGGGCGGCTCTATCCCGTCA-3’
	Reverse	5’-AAGGGCCAGAGGCGAACACG-3’
PDE9A	Forward	5’-GGCCCCGGAAAACACCACAGAG-3’
	Reverse	5’-AGTCTGTGCACACATTGAGATGTCC-3’
PDE10A	Forward	5’-CGATGCCTACGCGGACCCTC-3’
	Reverse	5’-TGCACCACGCCAATCACGCT-3’

[1] Das A, Xi L and Kukreja RC (2005) Phosphodiesterase-5 Inhibitor Sildenafil Preconditions Adult Cardiac Myocytes against Necrosis and Apoptosis. *Journal of Biological Chemistry,* **280,** 12944-12955.

[2] Kita K, Takahashi K, Ohashi Y, Takasuka H, Aihara E and Takeuchi K (2008) Phosphodiesterase Isozymes Involved in Regulation of Secretion in Isolated Mouse Stomach in Vitro. *Journal of Pharmacology and Experimental Therapeutics,* **326,** 889-896.

[3] Yang G, McIntyre KW, Townsend RM, Shen HH, Pitts WJ, Dodd JH, Nadler SG, McKinnon M and Watson AJ (2003) Phosphodiesterase 7A-Deficient Mice Have Functional T Cells. *The Journal of Immunology,* **171,** 6414-6420.

Supplementary Table 2. Apoptotic cell death in neural stem cell cultures following exposure to PDE5 inhibitors.

	Treatment	% Apoptotic death
6 h	Control	13.7 ± 1.4%
	1 µM T0156	9.5 ± 1.5% (n.s.)
	1 µM Sildenafil	16.7 ± 0.9% (n.s.)
	10 µM Zaprinast	19.3 ± 2.5% (n.s.)
24 h	Control	15.5 ± 1.0%
	1 µM T0156	13.9 ± 1.9% (n.s.)
	1 µM Sildenafil	16.8 ± 2.0% (n.s.)
	10 µM Zaprinast	10.6 ± 1.8% (n.s.)

Apoptotic cell death was assessed using the nuclear dye 7-AAD, detected by flow cytometry following treatment with PDE5 inhibitors for 6 h or 24 h. Data are expressed as means ± SEM of at least 6 independent experiments. One-way ANOVA (Dunnett´s post-test), $p>0.05$ (non-significant; n.s.), not different from control.

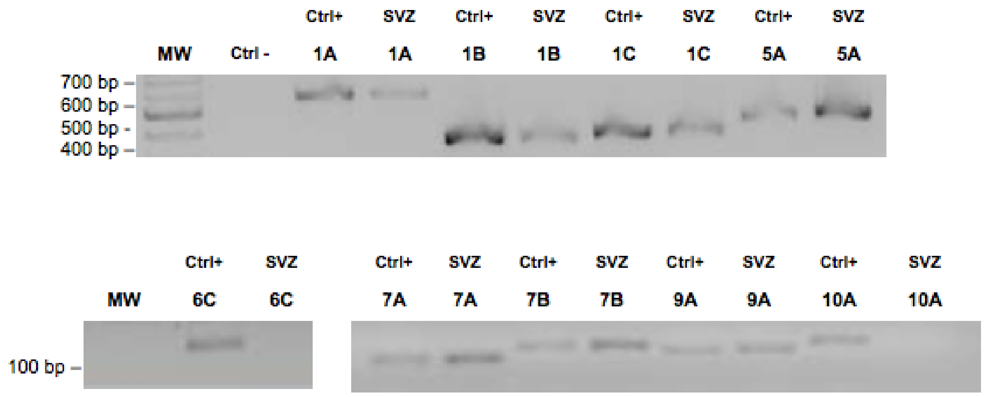

Supplementary Figure 1

Suppl. Fig. 1 Expression of PDE in SVZ cultures. mRNA expression of PDE1, 5, 6, 7, 9 and 10 in the mouse SVZ was assessed by PCR. mRNA from mouse brain was used as a positive control; for PDE6 the positive control used was mRNA from mouse retina

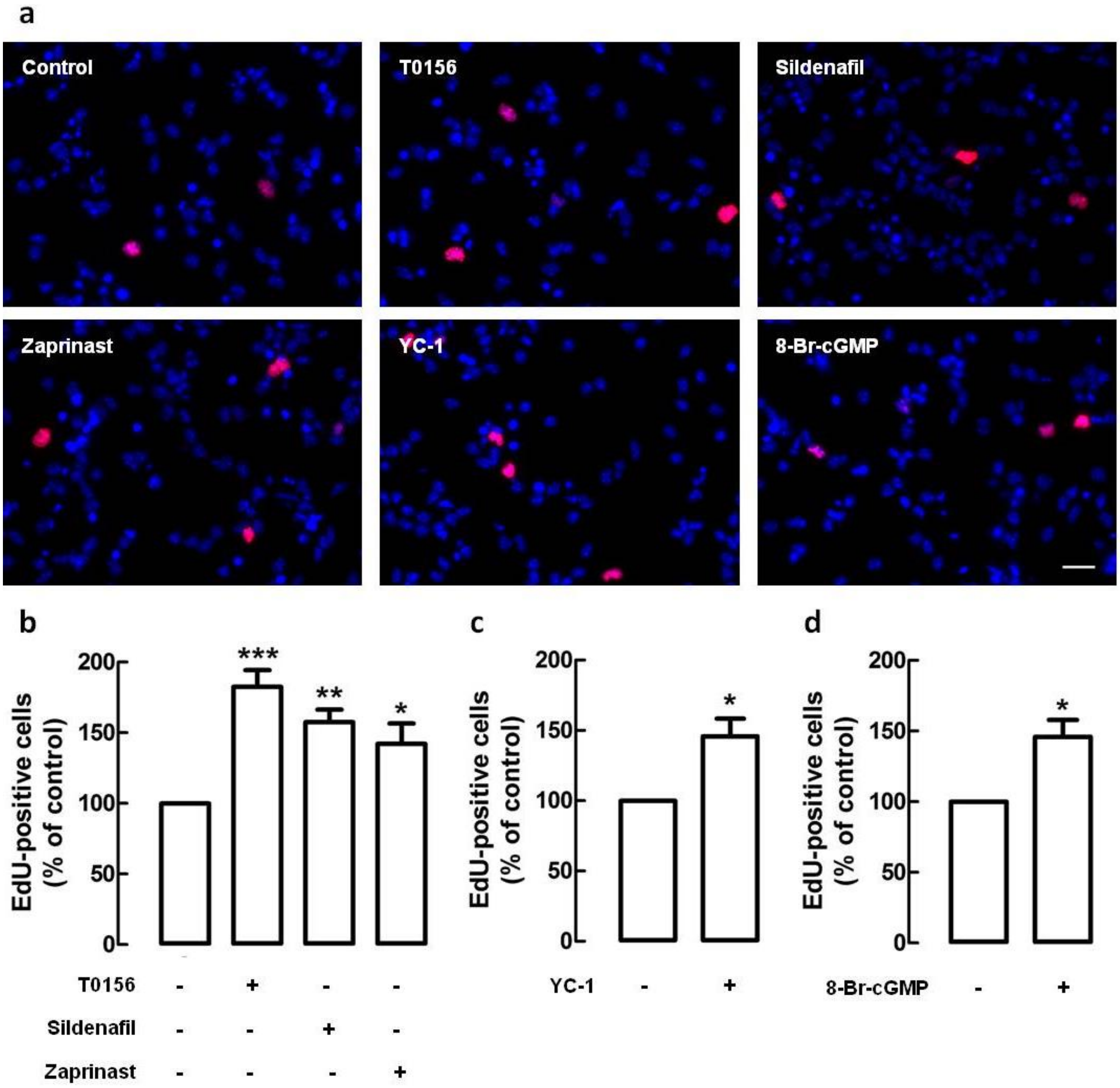

Supplementary Figure 2

Suppl. Fig. 2 The PDE5 inhibitors, the sGC activator and the cGMP analogue stimulated the proliferation of SVZ neural stem cells following 24 h of exposure. Cells were treated with the PDE5 inhibitors 1 µM T0156, 1 µM sildenafil, 10 µM zaprinast (**b**), or with the sGC activator, 20 µM YC-1 (**c**), or the cGMP analogue, 20 µM 8-Br-cGMP (**d**), for 24 h. The incorporation of EdU was assessed by fluorescence microscopy. Representative images of EdU-positive cells are shown (red) in neural stem cells after exposure to PDE5 inhibitors, YC-1 or 8-Br-cGMP for 24 h (**a**). Nuclei are labeled with Hoechst 33342 (blue). Scale bar: 20

µm. Data are expressed as means ± SEM of at least 4 independent experiments. (**b**) One-way ANOVA (Dunnett's post-test), *$p<0.05$, **$p<0.01$ and ***$p<0.001$, significantly different from control and, (**c**) and (**d**) two-tailed t test, *$p<0.05$, significantly different from control.

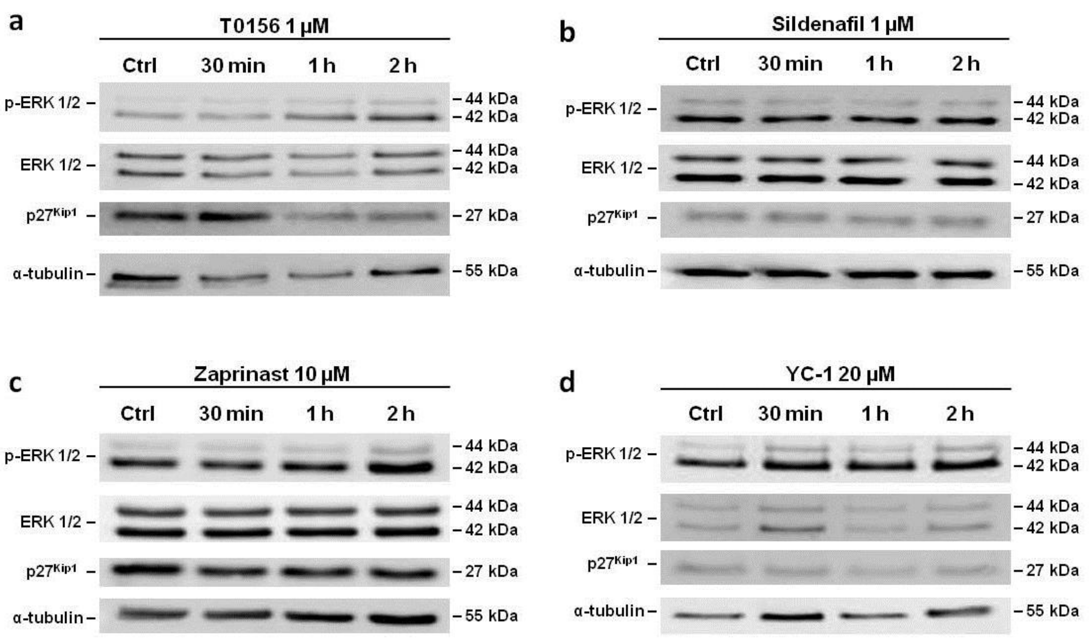

Supplementary Figure 3

Suppl. Fig. 3 PDE5 inhibitors increased signaling by the ERK1/2 pathway up to 2 h of exposure. SVZ stem cells were treated with 1 µM T0156 (**a**), 1 µM sildenafil (**b**), 1 µM zaprinast (**c**) and 20 µM YC-1 (**d**) for 30 min, 1h or 2h

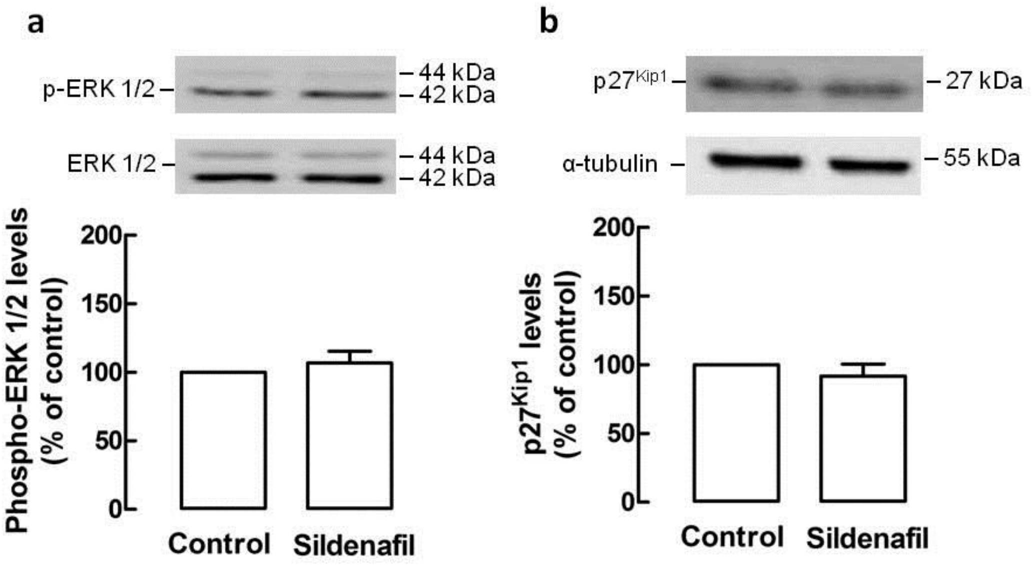

Supplementary Figure 4

Suppl. Fig. 4 Sildenafil did not alter the basal levels of phospho-ERK1/2 and p27^{Kip1} following 6 h of treatment. Phospho-ERK1/2 (**a**) and p27^{Kip1} levels (**b**) following treatment with 1 µM sildenafil for 6 h were assessed by Western blot. Data are expressed as means ± SEM of at least 3 independent experiments. Two-tailed t test ($p>0.05$)
